# Molecular alterations of the TLR4-signaling cascade in canine epilepsy

**DOI:** 10.1186/s12917-020-2241-x

**Published:** 2020-01-20

**Authors:** Eva-Lotta von Rüden, Fabio Gualtieri, Katharina Schönhoff, Maria Reiber, Fabio Wolf, Wolfgang Baumgärtner, Florian Hansmann, Andrea Tipold, Heidrun Potschka

**Affiliations:** 1 Institute of Pharmacology, Toxicology, and Pharmacy, Ludwig-Maximilians-University (LMU), Königinstr. 16, D-80539 Munich, Germany; 2 Department of Pathology, University of Veterinary Medicine Hanover, Buenteweg 17, D-30559 Hanover, Germany; 3 Clinic for small animals, University of Veterinary Medicine Hanover, Buenteweg 9, D-30559 Hanover, Germany

**Keywords:** Brain, Seizure, Inflammation, Toll-like receptor 4, HMGB1, HSP70, Idiopathic epilepsy, Structural epilepsy

## Abstract

**Background:**

Cumulating evidence from rodent models points to a pathophysiological role of inflammatory signaling in the epileptic brain with Toll-like receptor-4 signaling acting as one key factor. However, there is an apparent lack of information about expression alterations affecting this pathway in canine patients with epilepsy. Therefore, we have analyzed the expression pattern of Toll-like receptor 4 and its ligands in brain tissue of canine patients with structural or idiopathic epilepsy in comparison with tissue from laboratory dogs or from owner-kept dogs without neurological diseases.

**Results:**

The analysis revealed an overexpression of Toll-like receptor-4 in the CA3 region of dogs with structural epilepsy. Further analysis provided evidence for an upregulation of Toll-like receptor-4 ligands with high mobility group box-1 exhibiting increased expression levels in the CA1 region of dogs with idiopathic and structural epilepsy, and heat shock protein 70 exhibiting increased expression levels in the piriform lobe of dogs with idiopathic epilepsy. In further brain regions, receptor and ligand expression rates proved to be either in the control range or reduced below control levels.

**Conclusions:**

Our study reveals complex molecular alterations affecting the Toll-like receptor signaling cascade, which differ between epilepsy types and between brain regions. Taken together, the data indicate that multi-targeting approaches modulating Toll-like receptor-4 signaling might be of interest for management of canine epilepsy. Further studies are recommended to explore respective molecular alterations in more detail in dogs with different etiologies and to confirm the role of the pro-inflammatory signaling cascade as a putative target.

## Background

Over the last two to three decades, evidence has cumulated pointing to a key pathophysiological role of excessive inflammatory signaling in the epileptic brain [1, 2]. Experimental data from rodent models confirmed that enhanced activation of inflammatory pathways can contribute to enhanced excitability and lowered thresholds in the epileptic brain [3–5]. Moreover, increased expression rates of various pro-inflammatory mediators have been demonstrated in brain tissue from rodent epilepsy models as well as human patients with epilepsy [1, 6–10].

Among these mediators Toll-like receptor (TLR) signaling has been attributed a crucial role [2, 11]. As the best characterized ligand of TLR4, the danger associated molecular pattern molecule (DAMP) high mobility group box 1 (HMGB1) has been intensely studied in rodent models with induced seizures or spontaneous seizures [3, 12–17]. Enhanced release of HMGB1 proved to reduce seizure thresholds and increase seizure susceptibility [3, 16]. The effect of the HMGB1 disulphide isoform on excitability were mediated by activation of TLR4. Further support for an ictogenic property of HMGB1/TLR4 signaling came from a study, which did not only demonstrate an antiepileptogenic effect but also provided evidence for an anticonvulsant effect of an inactivating HMGB1 monoclonal antibody in two acute seizure models in mice [18]. In addition, earlier studies reported that TLR4 antagonists exert anticonvulsant effects in two different acute seizure models and a chronic epilepsy model [3]. Moreover, in comparison with wild-type mice TLR4-deficient mice develop less severe epilepsy following status epilepticus [19]. Thus, convincing evidence exists that targeting of TLR4-signaling pathways might be of particular interest for management of epilepsy based on disease-modifying approaches.

As we have previously discussed [20], further ligands of TLR4 receptors should be considered when developing strategies preventing TLR4-mediated increases in excitability and seizure susceptibility. In this context, it is of interest that the inducible heat shock protein 70 (HSP70) proved to be up-regulated in the hippocampus and parahippocampal cortex in a rat post-status epilepticus model with epilepsy manifestation following a latency period [20]. HSP70 has been functionally classified as a modulator of TLR4 function [21, 22]. Recently, we confirmed its relevance in a kindling model, in which mice overexpressing human HSP70 exhibited an increased seizure susceptibility with lowered thresholds and generalized seizure occurring early during the stimulation paradigm [23].

Canine epilepsy with different etiologies has been suggested as a natural animal model, which can serve as a translational bridge between testing in highly standardized rodent models and human clinical studies [24]. However, so far there is an evident paucity of information about detailed neuropathological alterations in canine epilepsy, which in particular applies for the question whether relevant inflammatory signaling also occurs in the canine epilepsy with different etiologies. Considering the prominent role of TLR4-signaling, we have focused this first study on the analysis of the distribution and expression rates of the TLR4 ligands HMGB1 and HSP70. Findings in dogs with structural epilepsy caused by identified cerebral pathology and idiopathic epilepsy were analyzed separately and compared. In addition, we assessed the impact of recent seizure clusters or status epilepticus in tissue from subgroups of dogs, which exhibited repetitive seizure patterns (at least two seizures per day = cluster) or beginning of continuous seizure activity during a time span between 1 h and 5 days before death.

## Results

### Clinical diagnosis

The first seizure event occurred in a time interval from 1 day to 11 years before the last clinical presentation prior to death or euthanasia. All dogs included in this study exhibited convulsive seizures (focal and generalized). In the anamnesis, there was no report about atonic, absence or myoclonic seizures. The seizure frequency varied in a wide range with dogs presenting only one seizure per month and others presenting seizure clusters resulting in up to 120 seizures per month.

The underlying lesions of dogs grouped with structural epilepsy covered a spectrum of four dogs with encephalitis, three dogs with a brain tumor, two dogs with hydrocephalus and one dog each with cerebral infarct, vacuolization of the frontal white matter and leucoencephalomalacia.

HE stained brain slices were examined to analyze seizure-induced secondary lesions in the hippocampus of epileptic animals. Morphological lesions in the hippocampus included a segmental vacuolation of the neuropil in four dogs, a gliosis in two dogs and a granulomatous inflammation in one dog. In 12 dogs, we did not detect significant cellular alterations.

For epileptic dogs with very high or very low protein expression we checked for an association between the time-gap between the last epileptic seizure and euthanasia/death. However, we did not find any coherence.

In addition, we analyzed if the kind of lesion (i.e. encephalitis versus brain tumor) had an effect on protein expression levels (TLR4, HMGB1, HSP70 and NeuN) within the group of structural epilepsy. Due to the low animal numbers within the subgroups, statistical analysis based on the kind of lesion was only possible for the encephalitis and tumor subgroup. Altogether, the kind of lesion did not affect protein expression for none of the analyzed proteins.

### Impact of epilepsy on the TLR4-signaling cascade in canine patients

**Fig. 1:**
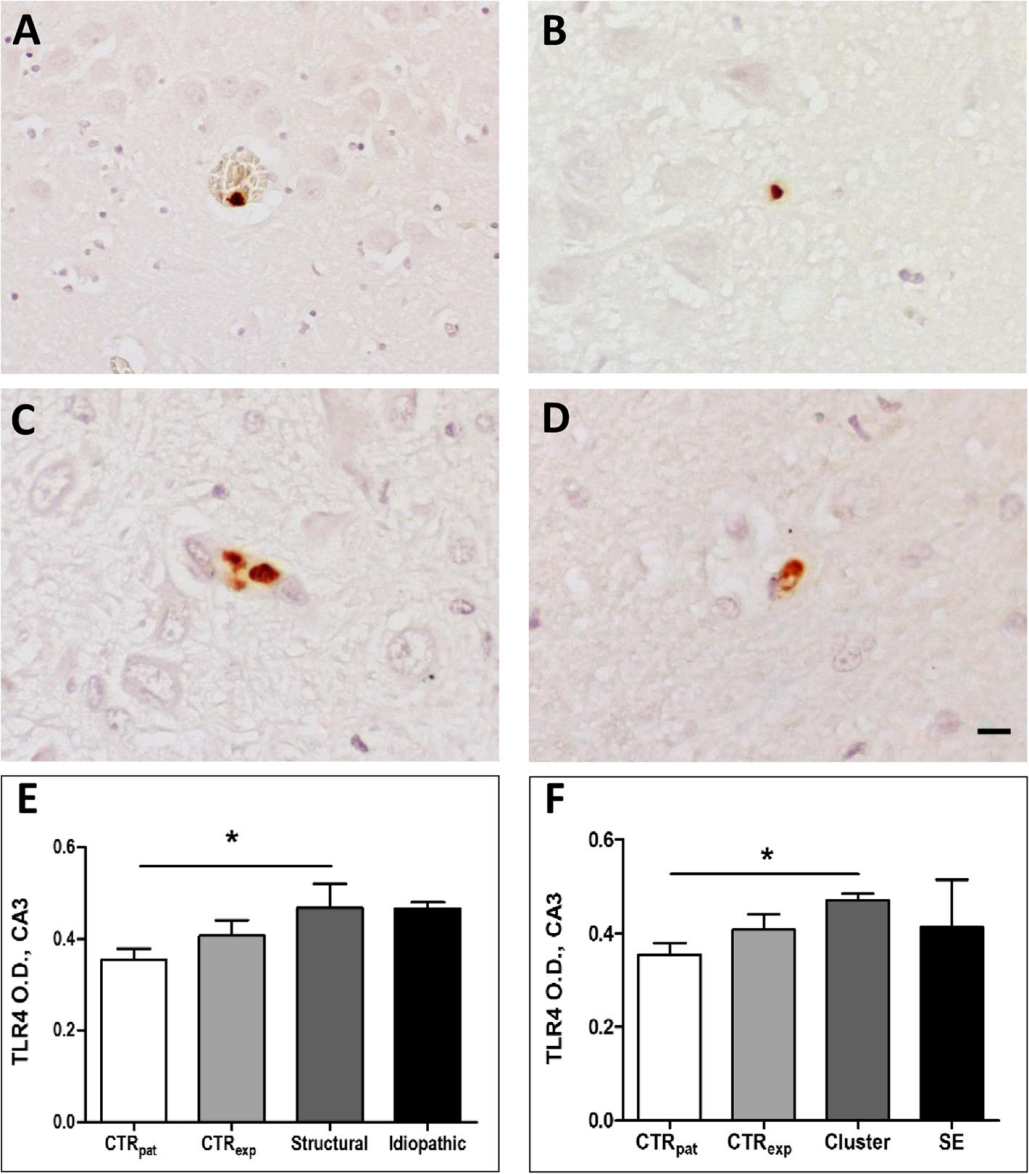
TLR4 expression in CA3. Hippocampal formation CA3 region representative microphotographs of TLR4-positive stained cells of dogs belonging to the patient control (**a**), experimental control (**b**), structural (**c**), and idiopathic group (**d**). TLR4 positive cells are often associated to blood vessels (**a**) and they might appear as single cells (**a**, **b**, **d**) or in clusters (**c**). Quantitative analysis of TLR4 expression (O.D.) in the CA3 region accordingly to epilepsy type (**e**) and seizure activity (**f**). *CTR*_*pat*_: patient control dogs; *CTR*_*exp*_: experimental control dogs; *Cluster*: dogs with cluster seizures; *Structural*: dogs with structural epilepsy; *SE*: dogs with status epilepticus; *Idiopathic*: dogs with idiopathic epilepsy. Scale bar 10 μm

We analyzed TLR4 expression (optical density (O.D.)) in the cornu ammonis region (CA) 1, CA3, dentate gyrus, hilus sub-region of the hippocampal formation, and in the piriform lobe by immunohistochemistry in canine brain tissue. In all sub-regions, we detected only very sparse positive cells that either appear as single cells or cell clusters. TLR4 positive cells were often associated to blood vessels or capillaries (see Fig. 1a-d). The intensity of TLR4 expression in animals with epilepsy was only altered in the CA3 sub-region of the hippocampus of dogs with structural epilepsy. The respective O.D. exceeded that in owner kept control dogs by 32% (F (3, 41) = 2.791, *p* = 0.0535; CTR_pat_ vs. Structural *p* < 0.05; see Fig. 1e). In contrast, TLR4 expression proved to be in the control range in dogs with idiopathic epilepsy. Our analysis of TLR4 expression levels did not reveal any significant group differences in any of the other brain regions (see Table 1).

**Table 1:** Statistical data of TLR4 O.D. (Type of epilepsy, statistical test: one-way ANOVA of variance)

Region	F-statistic (df1, df2)	*P*-value
CA1	2.048 (3, 42)	0.1216
DG	0.7244 (3, 40)	0.5434
Hilus	0.9539 (3, 41)	0.4236
Pir	1.027 (3, 30)	0.3944

**Fig. 2:**
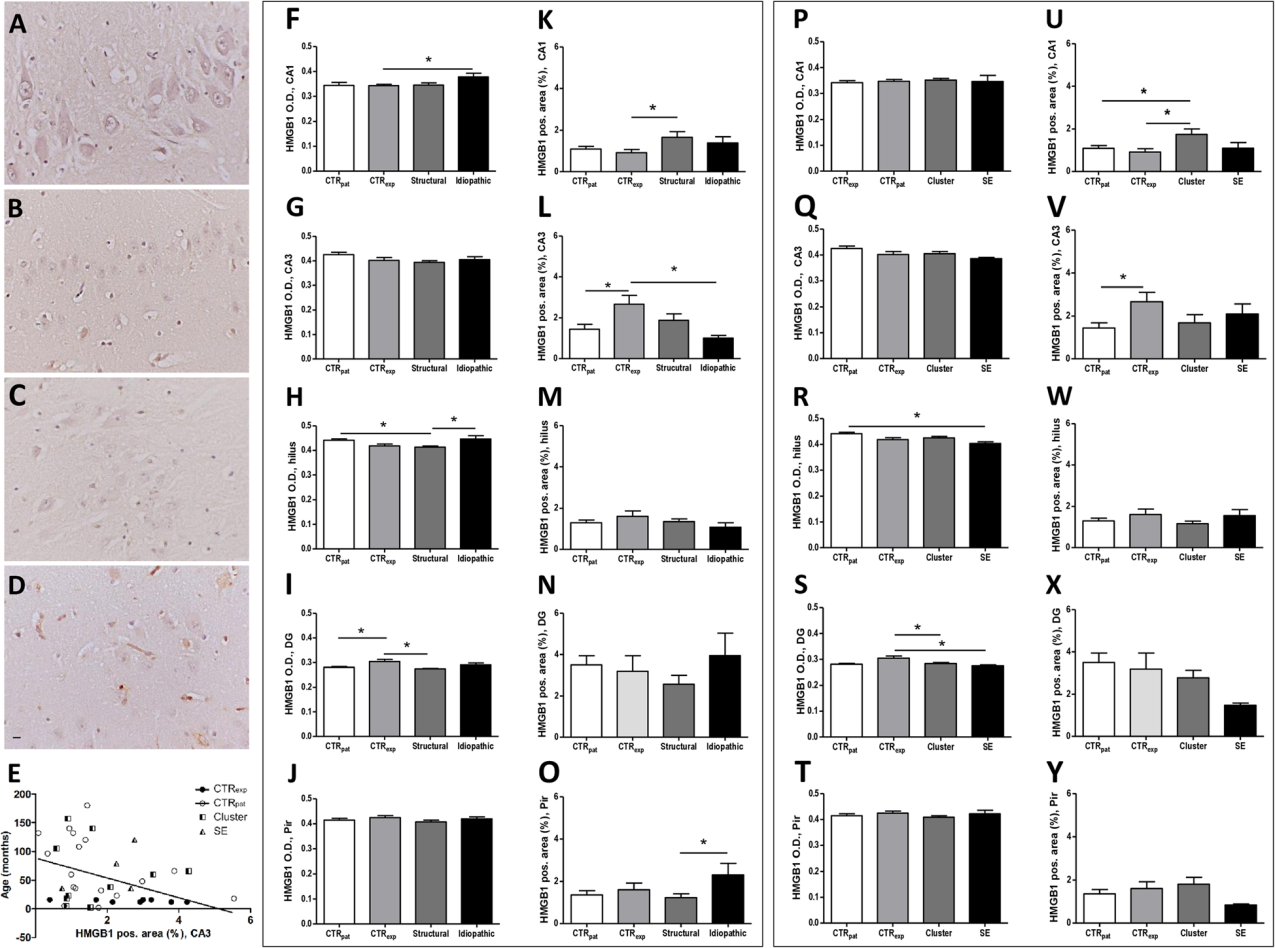
HMGB1 expression in all investigated areas. Representative histological images of HMGB1-positive stained cells in the CA1 region of the hippocampus of dogs of patient control (**a**), experimental control (**b**), structural (**c**), and idiopathic group (**d**). The majority of immunopositive cells are intensely stained and have the morphology of microglia cells. Cells with the morphology of neurons have a less intense staining appearing in the cytoplasm. Correlation analysis of HMGB1 positive area with age (**e**). Impact of epilepsy type on HMGB1 expression (O.D. and positive labeled area) in CA1 (**f**, **k**), CA3 (**g**, **l**), hilus (**h**, **m**), dentate gyrus (DG; **i**, **n**), and piriform lobe (Pir; **j**, **o**). Impact of seizure activity on HMGB1 expression in CA1 (**p**, **u**), CA3 (**q**, **v**), hilus (**r**, **w**), dentate gyrus (DG; **s**, **x**), piriform lobe (Pir; **t**, **y**). All data are given as mean ± SEM; *p* < 0.05 was considered statistically significant (*). *CTR*_*pat*_: patient control dogs; *CTR*_*exp*_: experimental control dogs; *Cluster*: dogs with cluster seizures; *Structural*: dogs with structural epilepsy; *SE*: dogs with status epilepticus; *Idiopathic*: dogs with idiopathic epilepsy. Scale bar 10 μm

In addition, we analyzed the expression (O.D. and positive stained area) of the TLR4 ligand HMGB1 in the hippocampus (CA1, CA3, dentate gyrus and hilus) and in the piriform lobe of dogs with epilepsy. The majority of immunopositive cells exhibited a round to elliptical shape with a diameter of 4–8 μm and an intense signal. These cells resemble the shape of microglia cells. A second positive stained cell type presented a less intense positive signal in the cytoplasm. Considering the morphology, these cells might be neurons (see Fig. 2a-d).

Quantitative analysis of HMGB1 expression tended to be increased in the CA1 region of the hippocampus, whereas in CA3, dentate gyrus and hilus HMGB1 expression levels tended to be reduced (see Fig. 2f, k, g, l, h, m, i, n, j, o).

Quantitative analysis of HMGB1 expression in the CA1 region confirmed an elevated O.D. in dogs with idiopathic epilepsy in comparison to control dogs (F (3, 42) = 2.186, *p* = 0.1051; CTR_exp_ vs. Idiopathic *p* < 0.05, see Fig. 2f). The HMGB1-positive area was increased by 81% in animals suffering from structural epilepsy when compared to control dogs (F (3, 43) = 2.553, *p* = 0.069; CTR_exp_ vs. Structural *p* < 0.05; see Fig. 2k).

In the CA3 sub-region, the HMGB1-positive area was reduced by 62% in dogs with idiopathic epilepsy when compared to experimental control dogs (F (3, 37) = 3.983, *p* = 0.0156, CTR_exp_ vs. Idiopathic *p* < 0.05; see Fig. 2 l). The interpretation of these data needs to consider that a significant difference exists between the two control groups, i.e. owner-kept dogs with neurological disease and experimental control dogs (− 46%; CTR_exp_ vs. CTR_pat_ *p* < 0.05, see Fig. 2l).

In the hilus and dentate gyrus of patients with structural epilepsy, the intensity of the HMGB1 staining proved to be reduced in comparison with one of the control groups (hilus: F (3, 41) = 5.605, *p* = 0.0028; CTR_pat_ vs. Structural *p* < 0.05 and dentate gyrus: F (3, 43) = 6.189, *p* = 0.0015; CTR_exp_ vs. Structural *p* < 0.05; see Fig. 2h and i). A direct comparison of dogs with structural and idiopathic epilepsy in the hilus revealed a higher staining intensity in the latter group (Structural vs Idiopathic *p* < 0.05; see Fig. 2h). An analysis of the HMGB1-immunopositive area in these regions did not reveal significant differences (hilus: F (3, 42) = 1.072, *p* = 0.3722; dentate gyrus: F (3, 43) = 0.7485, *p* = 0.5297; see Fig. 2m and n).

In the piriform lobe, the HMGB1-positive area in dogs with idiopathic epilepsy exceeded that in dogs with structural epilepsy by 88% (F (3, 36) = 2.09, *p* = 0.1204, Structural vs. Idiopathic *p* < 0.05, see Fig. 2o).

**Fig. 3:**
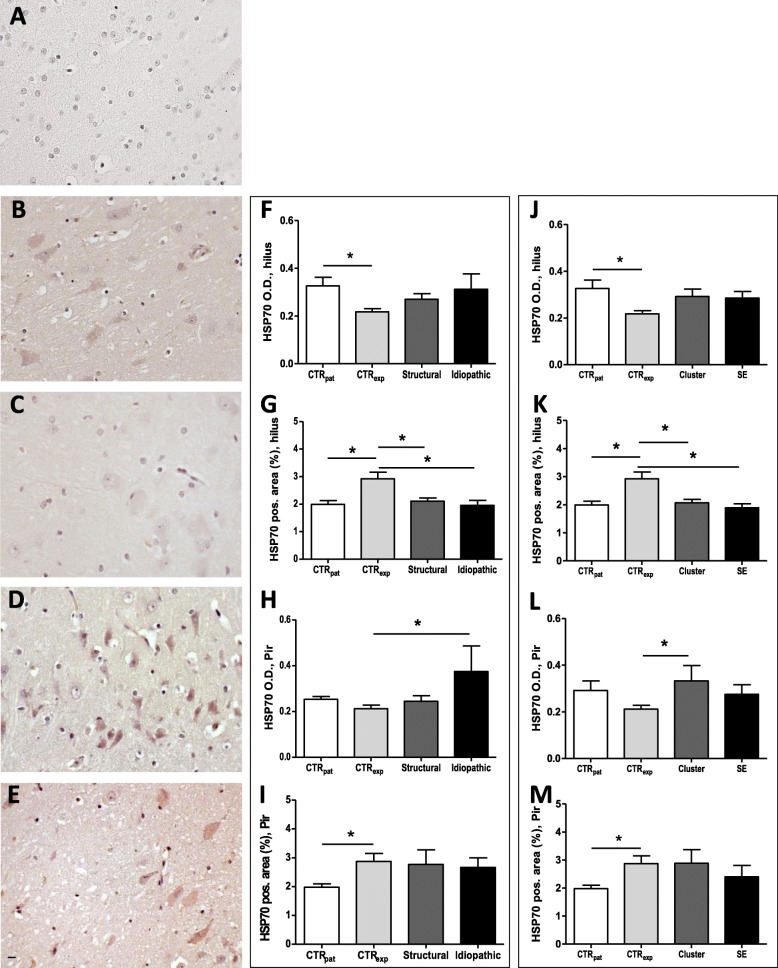
HSP70 expression in hilus and piriform lobe. Representative histological images of HSP70 negative control (**a**) and HSP70-positive stained cells in the piriform lobe of dogs of patient control (**b**), experimental control (**c**), structural (**d**), and idiopathic group (**e**). Immunopositive signal (in brown) was detectable from the cytoplasm of neuron-like shape cells. Impact of epilepsy type on HSP70 expression (O.D. and positive labeled area) in the hilus (**f**, **g**) and piriform lobe (**Pir; h**, **i**). Impact of seizure activity on HSP70 expression in the hilus (**j**, **k**) and piriform lobe **(Pir; l**, **m**). All data are given as mean ± SEM; *p* < 0.05 was considered statistically significant (*). *CTR*_*pat*_: patient control dogs; *CTR*_*exp*_: experimental control dogs; *Cluster*: dogs with cluster seizures; *Structural*: dogs with structural epilepsy; *SE*: dogs with status epilepticus; *Idiopathic*: dogs with idiopathic epilepsy. Scale bar 10 μm

In addition to HMGB1, we analyzed HSP70 expression in the hippocampus (CA1, CA3, dentate gyrus and hilus) and in the piriform lobe in brain tissue of dogs with chronic epilepsy. We observed immunopositive reactivity in the cytoplasm of cells with a neuronal morphology in all analyzed brain regions (see Fig. 3b-e).

Quantitative analysis of HSP70 expression (O.D. and HSP70-positive area) revealed only minor changes in epileptic dogs (see Fig. 3f-i). The most noticeable change of HSP70 expression was observed in the hilus with a significant reduction of the HSP70-positive area by 28 and 33% in animals with structural and idiopathic epilepsy, respectively (F (3, 43) = 6.661, *p* = 0.0009, CTR_exp_ vs. Structural and Idiopathic *p* < 0.05; see Fig. 3g). As the reduction might reflect neuronal loss in this region, we tested for a correlation between neuronal cell density and HSP70 expression considering data from control (CTR_exp_ and CTR_pat_) and dogs with epilepsy (Structural and Idiopathic). NeuN / HSP70 expression did not correlate in control animals (Pearson correlation coefficient *r* = 0.1661, *p* = 0.51), but there was a trend for a negative correlation in epileptic animals (Pearson correlation coefficient *r* = − 0.5275, *p* = 0.0526).

In the piriform lobe, the O. D of HSP70 was increased by 78% in animals with idiopathic epilepsy when compared to control animals (F (3, 24) = 3.584, *p* = 0.031, CTR_exp_ vs. Idiopathic *p* < 0.05; see Fig. 3h). For interpretation of these data, it needs to be taken into account that a direct comparison of the two control groups demonstrated a difference between data from both control groups (F (3, 26) = 1.703, *p* = 0.1943, CTR_exp_ vs. CTR_pat_ *p* < 0.05; see Fig. 3i). The analysis of correlation between HSP70 and NeuN expression in control (CTR_exp_ and CTR_pat_) and dogs with epilepsy (Structural and Idiopathic) indicated no correlation in CTR (Pearson correlation coefficient *r* = 0.5372, *p* = 0.0717) and a negative trend in dogs with epilepsy (Pearson correlation coefficient *r* = − 0.6915, *p* = 0.0852). In all other brain regions of interest, we detected no significant differences (see Tables 2 and 3).

**Table 2:** Statistical data of Hsp70 O.D. (Type of epilepsy, statistical test: one-way ANOVA of variance)

Region	F-statistic (df1, df2)	*P*-value
CA1	2.073 (3, 39)	0.1195
CA3	2.055 (3, 35)	0.1240
DG	1.717 (3, 40)	0.1790

**Table 3:** Statistical data of HSP70 immunopositive area (Type of epilepsy, statistical test: one-way ANOVA of variance)

Region	F-statistic (df1, df2)	*P*-value
CA1	1.138 (3, 40)	0.3452
CA3	0.9353 (3, 33)	0.4347
DG	1.576 (3, 39)	0.2106

### Impact of recent repetitive seizures and prolonged seizure activity on TLR4 signaling in canine patients

The TLR4 O.D. amounted to higher levels in dogs with cluster seizures as compared to control dogs (F (3, 39) = 2.408, *p* = 0.831; CTR_pat_ vs. Cluster *p* < 0.05; see Fig. 1f). In none of the other regions of interest, we identified significant group differences (see Table 4).

**Table 4:** Statistical data of TLR4 O.D. (Recent seizure activity, statistical test: one-way ANOVA of variance)

Region	F-statistic (df1, df2)	*P*-value
CA1	1.397 (3, 42)	0.2569
DG	0.5414 (3, 40)	0.6567
Hilus	1.009 (3, 40)	0.3989
Pir	0.9863 (3, 30)	0.4124

In the CA1 region the HMGB1-positive area was increased in animals with cluster seizures (F (3, 42) = 3.522, *p* = 0.0237; CTR_exp_ vs. Cluster and CTR_pat_ vs. Cluster *p* < 0.05, see Fig. 2u). Analysis of HMGB1 staining intensity and labelled area in CA3 did not confirm relevant group differences when comparing animals with epilepsy with the control groups (F (3, 38) = 2.008, *p* = 0.1307; see Fig. 2q). In this region, the HMGB1-positive area negatively correlated with age in control animals (Pearson correlation coefficient *r* = − 0.4529, *p* = 0.023, see Fig. 2e), but not in patients with epilepsy. In the hilus, we found a reduced O.D. in dogs with status epilepticus (− 9%; F (3, 39) = 5.025, *p* = 0.0052; CTR_exp_ vs. SE *p* < 0.05¸ see Fig. 2r). Moreover, in the dentate gyrus, the O.D. decreased by 7 and 10% in dogs with cluster seizures and in dogs with status epilepticus, respectively (F (3, 42) = 4.828, *p* = 0.006, CTR_pat_ vs. Cluster and CTR_pat_ vs. SE *p* < 0.05; see Fig. 2s). In both regions, the HMGB1-positive area proved to be in the control range (hilus: F (3, 41) = 1.262, *p* = 0.3013, dentate gyrus: F (3, 41) = 1.235, *p* = 0.3103; see Fig. 2w, x). To analyze if the reduced HMGB1-positive area might reflect neuronal loss in this region, we tested for a correlation between the number of neurons and the HMGB1-positive area. HMGB1 expression did neither correlate with neuronal density in control animals (CTR_exp_ and CTR_pat_: Pearson correlation coefficient r = − 0.08606, *p* = 0.7426) nor in animals with epilepsy (Structural and Idiopathic: Pearson correlation coefficient r = 0.08736, *p* = 0.7766). In the piriform lobe, we detected no differences in HMGB1 expression (O.D.: F (3, 36) = 0.862, *p* = 0.4705, HMGB1-positive area: F (3, 34) = 1.094, *p* = 0.3662; see Fig. 2t, y).

Whereas the O.D. of HSP 70 expression was unaffected by recent seizure activity in the hilus (F (3, 42) = 1.848, *p* = 0.1544, see Fig. 3j), the HSP70-immunopositive area proved to be reduced in dogs with cluster seizures and status epilepticus (F (3, 42) = 6.946, *p* = 0.0007, CTR_exp_ vs. Cluster and CTR_exp_ vs. SE *p* < 0.05; see Fig. 3k). In the piriform lobe of animals with recent cluster seizures the HSP70 staining intensity exceeded that in control animals (F (3, 26) = 1.587, *p* = 0.2198, CTR_exp_ vs. Idiopathic *p* < 0.05; see Fig. 3l). In all other regions (CA1, CA3 and dentate gyrus), recent seizure activity remained without impact on HSP70 expression (see Tables 5 and 6). It is emphasized that differences in HSP70 expression became evident between both control groups in different brain regions (F (3, 42) = 1.848, *p* = 0.1544, CTR_pat_ vs. CTR_exp_ *p* < 0.05; see Fig. 3j; F (3, 42) = 6.946, *p* = 0.0007, CTR_pat_ vs. CTR_exp_ *p* < 0.05; see Fig. 3k; (3, 26) = 1.968, *p* = 0.1469, CTR_pat_ vs. CTR_exp_ *p* < 0.05 see Fig. 3m).

**Table 5:** Statistical data of HSP70 O.D. (Recent seizure activity, statistical test: one-way ANOVA of variance)

Region	F-statistic (df1, df2)	*P*-value
CA1	1.705 (3, 39)	0.1818
CA3	1.875 (3, 34)	0.1524
DG	1.601 (3, 39)	0.2047

**Table 6:** Statistical data of HSP70 immunopositive area (Recent seizure activity, statistical test: one-way ANOVA of variance)

Region	F-statistic (df1, df2)	*P*-value
CA1	0.8182 (3, 39)	0.4917
CA3	0.8163 (3, 32)	0.4944
DG	1.373 (3, 38)	0.2656

Considering differences in the age range between the control groups, we tested whether HSP70 O.D. correlates with age. The respective analysis did not identify a correlation between these parameters (Pearson correlation coefficient CA1: r = 0.3565, *p* = 0.0738; CA3: *r* = 0.2684, *p* = 0.1759; dentate gyrus: *r* = 0.2891, *p* = 0.1435 and hilus: *r* = 0.2713, *p* = 0.1711).

### Neurodegeneration in hippocampal CA1 and hilus

**Fig. 4:**
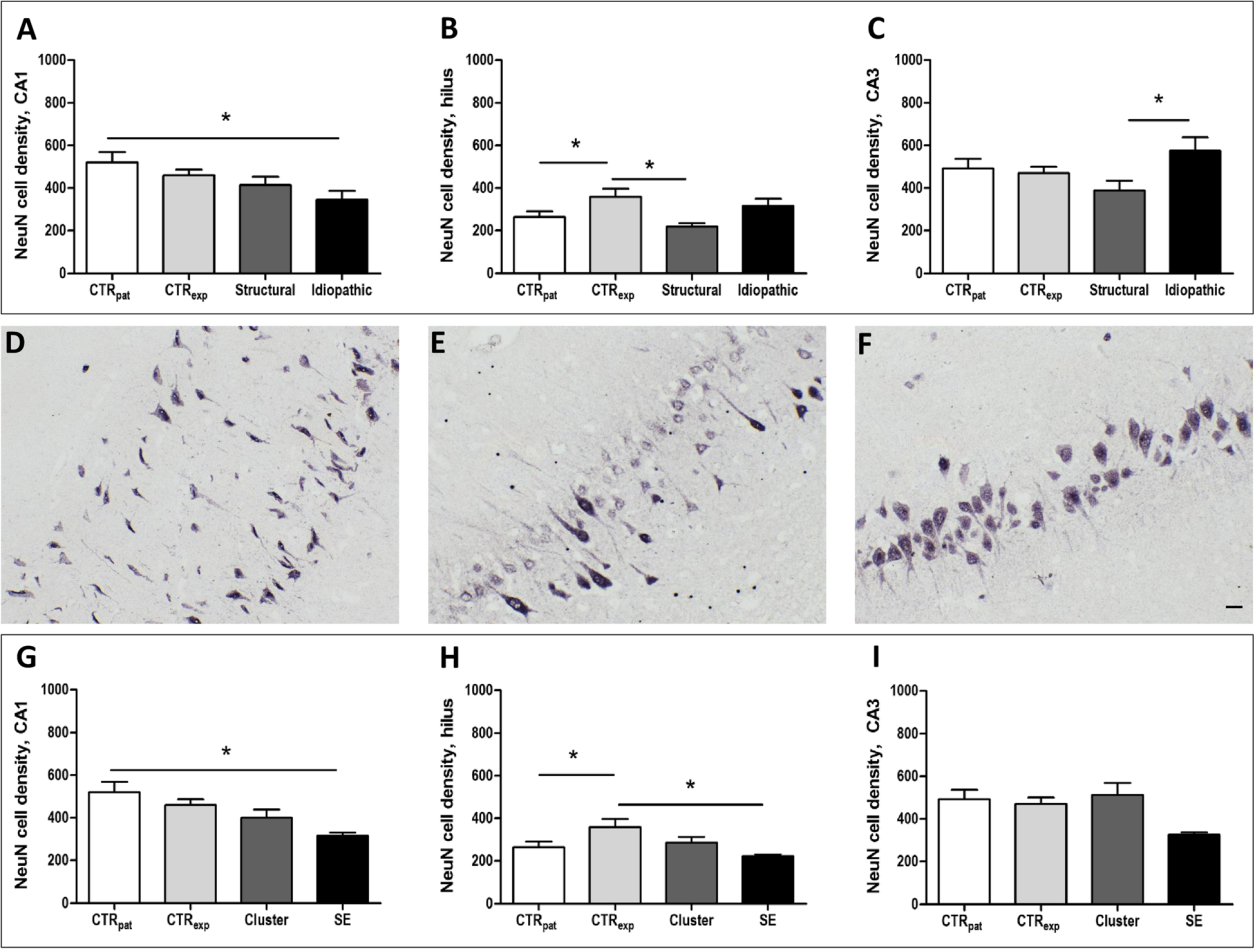
Neurodegeneration in CA1 and hilus. Analysis of NeuN cell density (number of cells per mm^2^) in the CA1 (**a**), hilus (4**b**) and CA3 (**c**) of the hippocampal region considering the type of epilepsy. All data are given as mean ± SEM; *p* < 0.05 was considered statistically significant (*). Hippocampal formation CA1 region representative microphotographs of dogs belonging to the patient control (**d**), structural (**e**) and idiopathic group (**f**). Analysis of NeuN cell density (number of cells per mm^2^) in the CA1 (**g**), hilus (**h**) and CA3 (**i**) of the hippocampal region considering recent seizure activity. All data are given as mean ± SEM; *p* < 0.05 was considered statistically significant (*). *CTR*_*pat*_: patient control dogs; *CTR*_*exp*_: experimental control dogs; *Cluster*: dogs with cluster seizures; *Structural*: dogs with structural epilepsy; *SE*: dogs with status epilepticus; *Idiopathic*: dogs with idiopathic epilepsy. Scale bar 25 μm

We assessed neurodegeneration in the CA1, CA3, hilus sub-region of the hippocampal formation and in the piriform lobe based on NeuN immunolabeled sections (see Fig. 4).

In the CA1 region, the neuronal cell density was reduced by 34% in dogs suffering from idiopathic epilepsy (F (3, 31) = 2.837, *p* = 0.0561; CTR_pat_ vs. Idiopathic *p* < 0.05; see Fig. 4a) when compared to owner kept dogs without central nervous system diseases. Moreover, the neuronal cell density was reduced in the hilus of patients with structural epilepsy by 39% (F (3, 33) = 4.476, *p* = 0.0103; CTR_pat_ vs. Structural *p* < 0.05; see Fig. 4b). Surprisingly, significant differences in hilar neuronal cell counts were also observed between both control groups (27%, (3, 33) = 4.476, *p* = 0.0103; CTR_exp_ vs. CTR_pat_ *p* < 0.05; see Fig. 4b).

Counts of NeuN- immunopositive cells remained unaffected in epileptic animals in CA3 (F (3, 33) = 2.589, *p* = 0.0713; see Fig. 4c) and in the piriform lobe (F (3, 24) = 2.031, *p* = 0.1404). However, when we directly compared dogs with structural and idiopathic epilepsy, the cell density in the CA3 region reached significantly higher levels in the latter group (F (3, 33) = 2.589, *p* = 0.0713; Structural vs. Idiopathic *p* < 0.05; see Fig. 4c).

It is known that the amount of neurons change with age in dogs [25, 26]. As the age of the two control groups differed significantly (F (3, 47) = 4.815, *p* = 0.0055; CTR_exp vs._ CTR_pat_ *p* < 0.05), we assessed whether NeuN expression correlates with age in these dogs. In none of the regions NeuN expression exhibited a correlation with age (Pearson correlation coefficient CA1: *r* = − 0.2731, *p* = 0.2888; CA3: r = 0.003, *p* = 0.9909; hilus: *r* = − 0.1281, *p* = 0.6123; piriform lobe: *r* = − 0.023, *p* = 0.9377).

Finally, we analyzed the impact of acute seizure activity in patients with recent cluster seizures or a status epilepticus. Dogs with status epilepticus exhibited a reduced neuronal cell density in the CA1 region (− 39%; F (3, 30) = 3.089, *p* = 0.0434; CTR_pat_ vs. SE *p* < 0.05; see Fig. 4g) and in the hilus (− 38%; F (3, 32) = 2.754, *p* = 0.0604; CTR_exp_ vs. SE *p* < 0.05; see Fig. 4h) when compared to control animals.

## Discussion

Analysis of components of the TLR4-signaling cascade revealed different expression patterns in canine patients with idiopathic and structural epilepsy. Moreover, expression analysis in dogs with recent repetitive or long-lasting seizure events provided evidence for molecular alterations in TLR4-signaling.

In the brain of different species including humans and laboratory rodents TLR4 expression has been reported in microglia and astrocytes with microglia exhibiting higher expression rates [27]. In the canine brain TLR4 expression has so far only been analyzed in tissue surrounding intracranial meningiomas [28]. The authors described scattered TLR4-expressing macrophages in the area of the tumor-brain interface [28]. Here, we now report first data for TLR4 expression in canine patients with structural or idiopathic epilepsy as well as in dogs without neuropathological findings. Predominant expression in microglial cells in the canine brain suggests that the basal expression pattern seems to be similar to that reported for other species [27]. Comparison between groups indicated an induction of TLR4 expression in the hippocampal CA3 region of dogs with structural epilepsy, whereas TLR4 expression proved to be in the control range in dogs with idiopathic epilepsy. Higher TLR4 expression rates might thus constitute a long-term consequence of an initial epileptogenic insult resulting in development of structural epilepsy in canine patients. Increased expression levels might result in excessive inflammatory signaling in dogs with structural epilepsy taking into account that TLR4 activation results in enhanced generation and release of pro-inflammatory cytokines including interleukin-1β and tumor-necrosis factor α [29–31]. A series of previous studies have demonstrated in rodent models that both cytokines can contribute to enhanced excitability, thereby triggering ictogenesis [1, 11, 32–34].

HMGB1 acts as a danger associated molecular pattern molecule released from astrocytes and neurons that acts as one of the main ligands and activators of TLR4. Its regulation has been reported in hippocampal specimen from human patients with epilepsy related to different etiologies as well as in rodent models of epilepsy [13, 17, 35–37]. In the present study, we obtained evidence for an upregulation of HMGB1 expression in the CA1 region of dogs with idiopathic and structural epilepsy. The increase in O.D. versus labelled area in the groups with different epilepsy types, suggest that the upregulation is related to an increase in the expression rate per cell in idiopathic epilepsy and an expansion of the cell population expressing HMGB1 at levels above detection threshold in structural epilepsy. The fact that antagonism of HMGB1 exerted anticonvulsant and antiepileptogenic effects in various rodent studies [3, 16, 38, 39] suggests that increases in HMGB1 observed in the CA1 region might promote seizure generation in canine epilepsy. Thus, pharmacological targeting of HMGB1 might also be of interest for management and prevention of canine epilepsy.

In this context it needs to be taken into account that the induction proved to be limited to the hippocampal CA1 region, which contrasted with the reduction of HMGB1 expression observed in other brain regions of interest. Moreover, it has been described that the functional consequences of HMGB1 signaling largely depend on the molecular isoform of HMGB1 and the intracellular translocation [37, 40]. In this context, it is of particular interest that a recent study confirmed that the pathologic disulfide HMGB1 isoform might serve as a mechanistic biomarker for epilepsy development and early epilepsy manifestation in rodent models and patients [37]. Thus, it is of future interest to complete more detailed analysis applying techniques, which allow to study the ratio between HMGB1 isoforms in the brain tissue from dogs with epilepsy.

As mentioned above, HSP70 serves as another modulator of TLR4-associated signaling [21, 22], which proved to be up-regulated in a post-status epilepticus model in rats. Therefore, the overexpression of HSP70 observed in the piriform lobe of dogs with idiopathic epilepsy might trigger TLR4-associated inflammatory signaling in this brain region. Considering the key role of this signaling cascade with generation of pro-inflammatory cytokines contributing to excessive excitability, targeting of HSP70 function or expression is suggested as another anti-inflammatory therapeutic concept for management of canine idiopathic epilepsy. This conclusion is supported by recent findings from our group [23]. In this study genetic overexpression of human HSP70 in mice resulted in a higher seizure susceptibility [23]. Again, it needs to be taken into account that enhanced expression was only evident in one brain region. Therefore, multi-targeting approaches modulating different molecular mediators might be more efficacious as compared to specific targeting strategies affecting a sole pro-inflammatory mediator. This conclusion is in line with previous discussions about combined anti-inflammatory treatment concepts as a basis for efficacious disease-modifying concepts [41–43].

In contrast to the finding for idiopathic epilepsy, HSP70 expression proved to be unaffected or decreased in brain regions of dogs with structural epilepsy. This result suggests that modulation of HSP70 function or expression does not constitute a promising strategy for treatment of this epilepsy type in canine patients.

Taking into account that induction of repetitive occurrence of seizures as well as prolonged seizure activity in laboratory rodents can trigger very pronounced molecular alterations including induction of different inflammatory signaling molecules [20, 44–46], we have additionally assessed expression patterns in dogs with recent seizure clusters or status epilepticus. The respective canine patients either died during seizures or were euthanized as a consequence of intractable epilepsy or drug-refractory status epilepticus.

Analysis of TLR4 expression revealed an induction in the hippocampal CA3 region in both subgroups, i.e. dogs with recent seizure clusters and status epilepticus. Thereby the increase in patients with seizure clusters was rather related to an upregulation of expression rates per cell, whereas the increase in patients with status epilepticus seems to be associated with an expansion of the population of cells expressing TLR4 above control level. With regard to expression of TLR4 ligands, a difference to control was only evident in animals with cluster seizures with an upregulation of HMGB1 in the hippocampal CA1 region and of HSP70 in the piriform cortex.

These data indicate that induction of these TLR4 activators can further promote excessive inflammation as a consequence of cluster seizures. However, it needs to be considered that with the use of post mortem tissue one cannot distinguish exactly between the impact of the underlying chronic disease and its etiology on one hand and the impact of recent repetitive or prolonged seizure activity on the other hand. In a recent proteomic study in rats, we have reported an early induction of HMGB1 in the hippocampus and of HSP70 in the hippocampus and parahippocampal cortex as a consequence of an electrically-induced status epilepticus [20]. Moreover, a status epilepticus-associated increase in HMGB1 has been reported in various other models in rats and mice [16, 47, 48].

The lack of increased HMGB1 and HSP70 in tissue from dogs with status epilepticus, is in apparent contrast to the experimental findings suggesting relevant species differences. Moreover, it needs to be taken into account that experimental rodent studies are in general performed in a highly standardized manner, whereas a variety of factors can influence molecular expression rates in studies focusing on post mortem tissue from patients.

Along this line, immunohistochemistry studies in patient tissue are often limited by the lack of appropriate control tissue. Considering different confounding factors, we have used tissue from two control groups for comparison with the epilepsy groups. The first batch came from patients with exclusion of neurological symptoms and lack of any neuropathological alterations. In this group of patients, we considered that hypoxic events, which can occur during agony related to different causes of death or to euthanasia, can affect heat shock protein expression rates [49–51].

Thus, we have introduced a second control group with tissue from experimental dogs that have previously been used in parasitology research. In this group the lower age range and a putative impact of previous parasite exposure needs to be taken into account for any comparative evaluation. A limitation might be that we do not know if this has an effect itself. However, even if these dogs were used for experiments before, their brains were free of a neuropathology and there were no findings of a general pathology in the periphery. Furthermore, all of these dogs were euthanized and right afterwards dissected. Thus, interference of extended time between death and brain removal or an influence of an extended agony phase can be excluded. This is in apparent contrast to the patient control group with owner kept dogs. Of course, these dogs were also free of a neuropathology. However, not all of them have been euthanized and blood-brain-barrier permeability can be already affected by hypoxia during agony and this might have an impact on the expression levels of the analyzed proteins.

We expected to see differences between the two control groups due to the lack of standardization of the patient control group. This group might be the better control for a direct comparison with the epileptic animals. Nevertheless, we also wanted to explore the effect of standardization itself and the respective impact in direct comparison to the epilepsy groups. The considerations received confirmation by the fact that differences between these control groups became evident with different analysis including that of HSP70 expression. These differences need to be taken into account when comparing with tissue from dogs with epilepsy. In this context, we would like to point out that the increase in CA1 HMGB1-positive area in dogs with cluster seizures constituted the only difference evident in comparison with both control groups.

In this context, age should be considered as a putative confounding factor. The finding that age correlated negatively with HMGB1 expression in the hippocampal CA3 region, suggests that an influence of age should be taken into account for HMGB1 analysis. Surprisingly, the direction of correlation is in contrast to previous findings from aged rats [52] indicating that species differences might exist regarding age-related development of HMGB1 expression.

Molecular alterations analyzed based on labelled area can be affected by cell loss. In view of a decrease of HSP70 expression in some brain regions of canine patients, we therefore additionally analyzed the impact of epilepsy on neuronal cell density. Disease-associated neuronal cell loss became evident in the CA1 region of dogs with idiopathic epilepsy. This outcome is unexpected in view of the fact that hippocampal cell loss in pyramidal layers is considered a hallmark of structural epilepsy of different etiologies rather than a characteristic feature of idiopathic epilepsy [53, 54]. However, high seizure frequencies and frequent status epilepticus have been reported in some dog breeds with a high prevalence of idiopathic epilepsy [55]. These might have contributed to CA1 neuronal cell loss as also substantiated by reduced cell counts in dogs with recent status epilepticus.

In dogs with structural epilepsy, the difference was only evident in comparison with the experimental controls. Thus, this finding needs to be interpreted with some caution, although correlation analysis argued against age as a confounding factor for hilar neuronal cell density.

## Conclusions

In conclusion, expression analysis of TLR4 and its ligands revealed complex changes, which differ between epilepsy types in canine patients. The regional up-regulation of the receptor and its ligands suggests that different molecular alterations might cause enhanced TLR4-signaling in different brain regions. Taken together, the data indicate that multi-targeting approaches modulating TLR4-signaling might be of interest for management of different types of canine epilepsy. Further studies are recommended to explore respective molecular alterations in more detail in dogs with different etiologies of epilepsy and to confirm the role of the pro-inflammatory signaling cascade as a putative target.

## Methods

### Animals and tissues

In this study, brain tissue of 48 dogs with an age range of 2 months to 15 years has been collected and processed as described earlier [56]. A subgroup of dogs was previously used as experimental dogs in parasitology research by the Institute of Parasitology of the University of Veterinary Medicine Hanover, Germany. These dogs are Beagle dogs kept in groups indoors in environmentally controlled rooms. They had free access to partly roof-covered outside runs. Animals were fed an age appropriate commercial dog diet at a recommended rate, had free access to water and got rubber toys for environmental enrichment. The previously performed studies in these dogs by the Institute of Parasitology are not content of this study and all brain samples used for this study were taken post mortem. This group was included as a separate control group for comparison due to the fact that hypoxia in a final disease state and during agony may cause brain cell stress triggering heat shock protein expression regardless of the type of the disorder and cause of natural death in owner kept dogs.

In short: after the death of the dogs (owner kept dogs: euthanasia or natural death due to different underlying diseases; experimental dogs: euthanasia), the brains were removed from the skull and fixed in 10% formalin for 10 days. The brains were cut in blocks, embedded in paraffin wax and cut in transverse three μm sections. Sections were then mounted on positively charged microscope slides (Superfrost plus, Menzel-Gläser, Braunschweig, Germany). Every section contained the hippocampus in a range from #1360 to #1660 of the canine brain atlas [57]. Dogs were distributed to different groups: 1st *patient control* group *(CTR*_*pat*_*)* comprising owner kept dogs without central nervous system diseases (*n* = 18, age range 2–180 months; mean 70.67 ± 12.58); 2nd the *experimental control* group *(CTR*_*exp*_*)* with inclusion of dogs without central nervous system diseases (*n* = 10, age range 12–16 months; mean 14 ± 0.67) and 3rd epileptic animals grouped by the type of epilepsy defined by etiology as suggested by the international veterinary task force [58] in epileptic animals with *structural epilepsy* caused by identified cerebral pathology (*n* = 12, age range 30–140 months; mean 81.17 ± 12.29) and *idiopathic epilepsy*, subtype unknown cause and no identification of structural epilepsy (*n* = 8, age range 2.5–157 months; mean 51.94 ± 18.82) or by the occurrence of seizure *clusters* (*n* = 9, age range 80–140 months; mean 68 ± 15.63) or *status epilepticus (SE, n* = 4, age range 36–120 months; mean 67.75 ± 20.15) occurring in a time span of 1 h to 5 days before death.

The grouping of epileptic dogs was based on the clinical diagnosis (anamnesis, neurological examination and pathological evaluation).

In this study, we decided to have two control groups with a patient control group and an experimental control group, which is more homogenous regarding several characteristics (same breed, similar age, same exposures/similar environment, standardized food and water, etc.). In the latter group, a high level of standardization is reached. We were interested if the effect of standardization itself has an impact on the different protein expression levels and we therefore wanted to additionally compare the experimental group directly to the epilepsy groups. Following the 3R concept, we aimed to keep animal numbers as low as possible and therefore used brain tissue from dogs, which were used in different experiments before (see above).

### Immunohistochemistry – staining procedures

To analyze seizure-induced secondary lesions in the brains of epileptic animals a Hematoxylin and Eosin (HE) staining was performed according to standard procedures and the hippocampus was examined.

For HSP70, TLR4, HMGB1, and NeuN immunostaining, the paraffin-embedded brain sections were deparaffinized and rehydrated. Afterwards, we performed heat induced epitope retrieval with sodium citrate pH 6 at 80 °C in the water bath for 30 min (for HSP70: 20 min in the microwave at 760 W (Severin 900 + Grill, Severin, Sundern, Germany)). In the following, sections were rinsed three times in Tris-buffered saline containing 0.05% Tween-20 (P9416, Sigma-Aldrich, Darmstadt, Germany; TBST) in cuvettes. All subsequent steps, except the washing, were performed in a humidity chamber. For HSP70 immunohistochemistry, we additionally incubated the sections in 3% H_2_O_2_ in Tris-buffered saline (TBS, pH 7) for 15 min. The sections were blocked with 0.25% casein (Sigma-Aldrich, Darmstadt, Germany) in TBS and incubated over night at 4 °C with primary antibody dissolved in antibody diluent (TBS with 0.25% casein and 0.1% Tween-20, see Table 7). After three washing steps with TBST in cuvettes, sections were incubated with the respective secondary antibody (see Table 7) for 60 min at room temperature. For TLR4 and HMGB1 immunohistochemistry, we next incubated the sections in 1% H_2_O_2_ in methanol for 15 min. Following washing in TBST in cuvettes, sections were incubated either for 30 min in Streptavidin/HRP 1:1400 in TBS (AB_2337238, Cat # 016–030-084, Jackson/Dianova GmbH, Hamburg, Germany) for HSP70 immunohistochemistry or for 60 min in the VECTASTAIN ABC-Peroxidase Kit, Standard Kit (Vector Laboratories Cat# PK-4000, RRID:AB_2336818) 1:100 in TBST. Sections were rinsed two times in TBST and then in TBS. Subsequently, sections were exposed to 3,3′-diaminobenzidine for 30 min (0.05% 3,3′-diaminobenzidine (CN75, Carl Roth GmbH & Co. KG, Karlsruhe, Germany) and 0.01% H_2_O_2_) for HSP70 immunohistochemistry or to SIGMAFAST 3,3′-diaminobenzidine tablets (D4418-50SET, Sigma-Aldrich Chemie GmbH, Taufkirchen, Germany) dissolved in bi-distilled water for TLR4 and HMBG1 immunohistochemistry. We washed all sections two times in TBS and one time in distilled water and counterstained all sections with Hemalum solution acidic according to Mayer (Roth T865, Carl Roth, Karlsruhe, Germany). After an additional washing step in distilled water, differentiation was carried out for 15 min under running tap water and after a final washing step in distilled water, we air-dried the sections overnight and used Entellan® (107,960, Merck, Darmstadt, Germany) for cover slipping. For all stainings, we processed negative controls in parallel omitting the incubation with the primary antibody. Detailed information about primary and secondary antibodies with dilution factors and manufacturing company can be found in Table 7.

**Table 7:** Primary and secondary antibodies with dilution factor and manufacturing company

Primary antibody	Dilution	Manufacturer	Secondary antibody	Dilution	Manufacturer
monoclonal mouse anti-Hsp70/72; C92F3A-5	1:100	Enzo Life Sciences, Lörrach, Germany	Biotinylated goat-anti-mouse	1:200	Dianova / Jackson GmbH, Hamburg, Germany
monoclonal mouse anti-TLR4; sc-293,072	1:500	Santa Cruz Biotechnology, Heidelberg, Germany	Biotinylated goat-anti-mouse, Vector BA-9200	1:500	BIOZOL Diagnostica Vertrieb GmbH, Eching Germany
polyclonal rabbit anti-HMGB1; ab227168	1:100	Abcam, Cambridge, UK	Biotinylated goat-anti-rabbit, 111–065-003	1:500	Dianova / Jackson GmbH, Hamburg, Germany
monoclonal mouse anti-NeuN; MAB377	1:100	Millipore/ Merck Chemicals GmbH, Darmstadt, Germany	Biotinylated goat-anti-mouse	1:500	Vector Laboratories, Burlingame, California, USA

### Immunohistochemistry – image analysis and quantification

An operator unaware of the group assignment analyzed HSP70, HMGB1, TLR4, and NeuN expression in the CA1 and CA3, in the dentate gyrus, in the hilus of the hippocampal formation as well as in the piriform lobe. The operator captured up to three images per analyzed brain region at 200x (HMGB1, TLR4, and NeuN) and up to five images at 400x (HSP70) magnification with an Olympus BH2 microscope with a single chip charge-coupled device (CCD) color camera (Axiocam; Zeiss, Göttingen, Germany), and an AMD Athlon™ 64 Processor based computer with an image capture interface card (Axiocam MR Interface Rev.A; Zeiss, Göttingen, Germany). Images were analyzed by ImageJ [59] software (ImageJ v1.51, RRID:SCR_003070, NIH). Up to three (200x) / five (400x) visual fields (588.14 × 440.68 μm / 297.22 × 222.70 μm) were evaluated per region.

For the analysis of NeuN immunohistochemistry, a well-trained operator counted positive stained neurons manually as none of the automatic available options were applicable. The cell density was expressed as the number of cells per area of interest in mm^2^.

HMGB1 and HSP70 expression was analyzed by the positive stained area per analyzed visual field in percent. In addition, TLR4, HMGB1 and HSP70 were evaluated by O.D. analysis. For the analysis of O.D., the operator performed a calibration for grey values following the instructions from the website [60]. With the color deconvolution plug in (vector H-DAB) an 8-bit RGB image was generated and we used color 2 (brown) for further analysis. For each staining, we used slightly modified protocols to meet the optimal analysis conditions.

For the evaluation of HMGB1 immunohistochemistry thresholds were set manually (CA1: 0.27, CA3: 0.32, dentate gyrus: 0.23, hilus: 0.33 and piriform lobe: 0.32) by measuring one visual field per group. From the obtained values a mean was computed and applied for the analysis. The dentate gyrus and the CA1 region have been analyzed twice as the first thresholds (dentate gyrus: 0.17 and CA1 0.23) proved to be not strict enough. For the analysis of TLR4 immunohistochemistry, the automated Intermodes threshold method [61] and for HSP70, the automated Triangle threshold method [62] was applied for all regions. We computed means from all obtained values for the individual animals and used them for statistical analysis.

### Statistics

For the statistical analysis of group differences, we used GraphPad Prism 5.04 for Windows (GraphPad Prism Software, San Diego, USA). We analyzed group differences of the O.D., positive stained area and cell density by one-way analysis of variance followed by Bonferroni Multiple comparison test of selected pairs. We used the correlation analysis of Pearson to investigate an association of the neuronal cell density and HMGB1 expression and of HSP70 expression in the hilus as well as for age and NeuN, TLR4, HMGB1 and HSP70 expression. We applied the Grubbs’ test to detect significant outliers and considered a *p* value < 0.05 statistically significant. All descriptive statistics are expressed as mean ± SEM.

## Data Availability

The datasets used and/or analyzed during the current study are available from the corresponding author on reasonable request.

## Abbreviations

CA1, CA3: Cornu Ammonis region 1,3
CTR_exp_: Experimental control group
CTR_pat_: Patient control group
DAMP: Danger associated molecular pattern molecule
HMGB1: High mobility group box 1
HSP70: Heat shock protein 70
TLR: Toll-like receptor
